# 

**Published:** 1897-10

**Authors:** 


### CONTENTS.

### COMMUNICATIONS.

The Relation of the Chemistry and Bacteriology of the Stomach to Dental Caries .......... 469
How to Establish a Dental Practice .......... 490
How to Conduct a Dental Practice in an Agricultural District .......... 501
How to Conduct a City Practice .......... 507

### EDITORIAL.

Educational .......... 516
A List of Colleges .......... 517
Register of Dentists .......... 517
Report of the Committee on Necrology of the National Association of Dental Faculties .......... 518

